# Development of the Japanese Version of Pregnancy-Related Anxiety Questionnaire—Revised-2: Measurement and Psychometric Properties

**DOI:** 10.3390/healthcare11131935

**Published:** 2023-07-04

**Authors:** Ritsuko Shirabe, Hiroko Okada, Tsuyoshi Okuhara, Rie Yokota, Takahiro Kiuchi

**Affiliations:** 1 Department of Health Communication, Graduate School of Medicine, The University of Tokyo, Tokyo 113-8655, Japan; 2 Department of Health Communication, School of Public Health, Graduate School of Medicine, The University of Tokyo, Tokyo 113-8655, Japan; okadahiroko-tky@umin.ac.jp (H.O.); okuhara-ctr@umin.ac.jp (T.O.); yokotarie-tky@umin.ac.jp (R.Y.); tak-kiuchi@umin.ac.jp (T.K.)

**Keywords:** pregnancy-related anxiety, pregnant women, anxiety, Pregnancy-Related Anxiety Questionnaire—Revised-2, psychometric properties

## Abstract

Tools to evaluate pregnancy-specific anxiety are lacking in Japan. This study aimed to develop a Japanese version of the Pregnancy-Related Anxiety Questionnaire—Revised-2. After scale translation and cognitive interviews, we conducted a cross-sectional study among 120 ≥18-year-old, singleton (pregnant with one baby) Japanese women before 15 weeks of pregnancy, recruited from four facilities. A total of 112 women completed the questionnaires. We tested the internal consistency, measurement error and reliability, structural validity, measurement invariance across nulliparous and parous women, construct validity by calculating omega, standard error of measurement (SEM), intraclass correlation coefficient (ICC), confirmatory factor analysis (CFA), multigroup CFA, multitrait-scaling analysis, correlational analyses with other measurements, and *t*-test to compare nulliparous and parous groups. Omega was 0.90 for the total score. SEM was 3.4 and ICC was 0.76. The CFA revealed an optimal fit for the three-factor model based on the original scale. Multigroup CFA supported measurement invariance across the nulliparous and parous groups, and multitrait-scaling analysis revealed 100% scaling success. The correlation coefficients with other scales of childbirth anxiety and general anxiety were 0.70 and 0.24. The mean total score of the nulliparous women was higher than that of the parous women (34.5 vs. 30.3, *p* = 0.001). Therefore, the scale was determined to have good validity and reliability.

## 1. Introduction

Postnatal maternal mental health is a major issue worldwide, with the global pooled prevalence of postpartum depression as high as 18% [1]. Japan is no exception, as 10–20% of mothers become depressed after childbirth [1]. Although the prevalence varies between studies, approximately 20% of women experience depression- or anxiety-related symptoms already during pregnancy [2,3]. Prenatal anxiety has been the focus of multiple studies in recent years; for example, a systematic review reported that antenatal anxiety was associated with postpartum depression independent of prenatal depression [4]. Prenatal anxiety also seems to be a predictor of birth-related outcomes (e.g., preterm birth and low-birth-weight baby [5]), which is also an ongoing issue in Japan [6]. To support women with anxiety during their antenatal period in Japan, it is necessary to identify pregnant women in need of support and create a means to prevent adverse birth outcomes and the deterioration of their mental health.

Among the several different anxiety types, pregnancy-related anxiety (PrA) is characterized specifically for pregnant women, and is defined as nervousness and fear in the context of pregnancy, childbirth, and parenting, accompanied by excessive worry and somatic symptoms [7]. PrA is a distinctive syndrome that is different from general indices of anxiety [8], with only low-to-moderate correlations with the general anxiety score [9]. Regarding predictive ability, PrA has been reported to have strong risk factors for adverse obstetric outcomes (e.g., preterm birth) [10], while this association is not consistent for general anxiety [9]. PrA has also been reported to predict postpartum affective disturbances, such as parenting stress and anxiety disorders [11,12], which often coexist with or lead to the development of depressive symptoms in the future [13,14].

The Pregnancy-Related Anxiety Questionnaire—Revised-2 (PRAQ-R2) is a reliable and validated scale for assessing PrA regardless of parity [15]. This scale can assess prenatal anxiety about child health, child loss, and childbirth, as well as anxiety about body, image which is unique among existing PrA scales [7]. Previous studies that assessed PrA with the PRAQ-R2 or the PRAQ-R (the previous version of PRAQ-R2) [8] have reported the association with various adverse outcomes such as delivery time [16], increased medical intervention [17], postpartum parenting stress in nulliparous women [12], and behaviors during pregnancy such as weight control and smoking [18]. There are some validated Japanese scales which can assess general anxiety (e.g., Hospital Anxiety and Depression Scale [19] and the General Health Questionnaire [20]), or specialized in some elements of PrA such as fear of childbirth (e.g., the Wijma Delivery Expectancy/Experience Questionnaire [21]). However, although PrA has been reported to be linked to negative outcomes, no existing scale can specifically measure multifaceted PrA in Japan.

This study aimed to develop an instrument for measuring multifaceted anxiety during early pregnancy in Japanese women for the first time by conducting a validity study for the Japanese version of the PRAQ-R2. After development, this scale is intended for research use in exploring the processes that predict psychological and physical outcomes and for clinical use in screening women in particular need of assistance to support them from the early stage of pregnancy.

## 2. Materials and Methods

This study followed the recommendations of the COnsensus-based Standards for the selection of health Measurement INstruments (COSMIN) study design checklist for patient-reported outcomes (PRO) measurement instruments [22]. This study was approved by the Institutional Review Board of the University of Tokyo (approval code: 2020154NI), and the participants’ signed informed consent was obtained before participating in the interview or answering the questionnaires.

### 2.1. Pregnancy-Related Anxiety Questionnaire—Revised-2 (PRAQ-R2)

This scale is based on the Pregnancy-Related Anxiety Questionnaire (PRAQ) [23]. The original Finnish version has been translated into various languages and widely used in recent years [24,25]. This is a 10-item self-report measure of reflective model with three factors identified by exploratory factor analysis in the original study [15]: fear of giving birth (FOGB), worries about bearing a physically or mentally handicapped child (WBHC), and concern about own appearance (COA). Items are rated from 1 (absolutely not relevant) to 5 (very relevant), with a higher total score indicating greater anxiety. The distinctiveness of PrA from general anxiety was assessed using the PRAQ-R [8] and subsequent validation studies in other languages [24,25].

### 2.2. Translation Process

The PRAQ-R2 was translated into Japanese following the principles of good practice for the translation and cultural adaptation process for PRO measures [26]. After obtaining permission to use the PRAQ-R2 from the developer, the English version of the PRAQ-R2 was translated into Japanese (forward translation) by two independent native Japanese translators (one had translation experience in the field of obstetrics and gynecology and the other did not). Next, R.S. (the first author) carried out the reconciliation of two forward translations into a single translation with H.O. (the second author, a Japanese native speaker residing in Japan, fluent in English, with a medical background and experience in translating a PRO measure). This reconciled translation was translated into English (back translation) by a translator naïve to the measured construct. Subsequently, a professional translator team reviewed the translation after R.S. explained the concept and constructs of the scoring scale. They found almost no differences in expression between the original English and reverse English translations, suggesting only minor changes to the natural Japanese flow. R.S., H.O. and other members of the research team reviewed the report and harmonized the new translation to ensure conceptual equivalence with the original and all versions of translations, referencing one difficult item (item 5) to the developer for clarification. Item 5 in the original version asked pregnant women if they felt they had an “unattractive appearance”. The reconciled translation used the expression “not attractive” in Japanese, but this seemed too aggressive for Japanese women. Considering the developer intended for this item to evaluate how women view their appearance (for instance in the mirror), we changed this to “not looking good” in Japanese while maintaining the original meaning as much as possible.

### 2.3. Content Validity

#### 2.3.1. Expert Meetings

To assess content validity from the perspective of professionals, we conducted two group meetings: one in an academic setting (with ten social medicine researchers, including medical doctors and nurses) in May 2020 and one in a medical setting (with four midwives) in June 2020. Both meetings were audio-recorded and lasted approximately 45 min. R.S. transcribed the audio recordings verbatim immediately after the meetings.

#### 2.3.2. Pilot Study through Cognitive Interviews

To assess content validity from the perspective of the patient, we conducted cognitive interviews with six pregnant women recruited at three facilities in Tokyo, Japan (a group interview with five women in November 2020 and an individual interview in December 2020). We used convenience sampling to rapidly recruit participants for the following validation study. Participant characteristics are listed in Table S1. Following the established focus group interview procedures [27], we designed interview guidelines based on semistructured questions (Table S2). The interviews were audio- and video-recorded and lasted for approximately 40 min. R.S. transcribed the audio recordings verbatim immediately after the interviews.

### 2.4. Validation Study

#### 2.4.1. Settings and Participants

This cross-sectional study was performed at four facilities in an urban area of Japan (two hospitals, one of which is a regional perinatal center, and two clinics). Outpatients who were Japanese, aged 18 years or older, singleton (pregnant with one baby), in early pregnancy (<15 weeks), and able to read Japanese were eligible for this study and consecutively recruited. We planned to recruit 120 women, taking into account the expected number of missing values, as >100 participants is sufficient to evaluate the measurement of properties of PRO measures in the COSMIN checklists [22]. Participants were recruited orally and in writing during regular checkups by the first author or research collaborators at each facility. They were informed that participation was voluntary and instructed to return the questionnaires either directly or by mail. The data were collected between April 2022 and January 2023. We sent the participants a JPY 200 (roughly equivalent to USD 2) gift certificate in return for completion of the questionnaire.

#### 2.4.2. Measures

In the questionnaire, participants were asked about their jobs, academic background, and annual household income. Age, marital status, and obstetric data (parity, previous abortion, means of conception, gestational age, height, and weight before pregnancy) were obtained from medical records. In addition, we asked the participants to respond to additional measures in the same questionnaires to evaluate the hypotheses for construct validity.

##### Prenatal Self-Evaluation Questionnaire (PSEQ)

No gold standard exists for this scale in Japan. The J-PSEQ is a validated and reliable instrument [28] which measures psychosocial adaptation to pregnancy and delivery and was translated from the original scale [29]. In this study, we used two subscales of J-PSEQ: “concern for well-being of self and baby” (nine items) and “fear of pain, helplessness, and loss of control” (ten items). Items were rated on a four-point Likert scale ranging from 1 (strongly disagree) to 4 (strongly agree), with higher total scores indicating greater anxiety about childbirth.

We used this scale to assess the convergent validity of the PRAQ-R2 as it was expected to have strong positive correlations with the sum of the two subscales of the PRAQ-R2 (FOGB and WBHC).

##### Hospital Anxiety and Depression Scale—Anxiety (HADS-A)

To examine discriminant validity, the Japanese version of the HADS-A was used for general anxiety assessment [19]. The factor structure and measurement variance of HADS-A were examined in a population of pregnant Japanese women [30]. HADS-A consists of seven items rated on a four-point Likert scale ranging from 1 (strongly disagree) to 4 (strongly agree). Higher scores indicate greater levels of general anxiety.

We expected a low-to-moderate positive correlation between the total PRAQ-R2 and HADS-A scores.

#### 2.4.3. Data Analysis

First, we calculated the mean values with standard deviations (SDs) for the sum scores and each subscale of the PRAQ-R2. The frequency distribution of each item was also examined.

##### Internal Consistency

We checked the item–total correlation and calculated the omega total value [31] for the total scale and subscale, and each scale after it was removed. Omega total values greater than 0.7 were acceptable.

##### Measurement Error and Reliability

We asked the participants to answer the PRAQ-R2 (Japanese version) approximately three days after the initial questionnaire when they felt calm and then to mail it. To evaluate the measurement error of the total PRAQ-R2 score, we calculated the standard error of measurement (SEM) and smallest detectable change (SDC) between the two time points. The intraclass correlation coefficient (ICC) was also calculated for the total score between the two time points to evaluate the reliability using a two-way mixed-effects model for absolute agreement.

##### Structural Validity

To test the three-factor structure proposed in the original scale, we calculated the polychoric correlations for each item. Next, we performed a confirmatory factor analysis (CFA) using the weighted least squares means and variance adjusted (WLSMV) estimator [25], which is based on standardized polychoric matrices and is more reliable for ordered variables (e.g., the Likert scale) than the popular maximum likelihood method [32,33]. Acceptable factor loadings were at least 0.5 [34]. We checked the following indices to evaluate the model fit: comparative fit index (CFI), Tucker–Lewis index (TLI), and root mean square error of approximation (RMSEA). CFI and TLI >0.95 indicates a good fit while >0.90 indicates an optimal fit [35]. RMSEA <0.06 indicates a good fit and <0.08 indicates an optimal fit [35].

##### Measurement Invariance across Groups

We performed a multigroup confirmatory factor analysis (MGCFA) across groups of nulliparous and parous samples. We applied three measurement invariance models with increasing cross-group restrictions: (1) a configural invariance model, in which the loading pattern was similar in both groups, but the magnitude of factor loadings, intercepts, and residual variances varied; (2) a weak invariance model, in which only the factor loadings were constrained to be equal; and (3) a strong invariance model, in which factor loadings and item intercepts were constrained to be equal. We calculated the decrease in model fit to compare the models. Change in scaled CFI (ΔCFI) ≤|−0.005| and change in scaled RMSEA (ΔRMSEA) ≤0.010 indicate measurement invariance between the two competing models, according to Chen’s suggestion for sample size with each group smaller than 300 [36].

##### Hypotheses Testing for Construct Validity

We performed a multitrait-scaling analysis to compare the subscales of the PRAQ-R2. Convergent validity was supported if there was a correlation (r ≥ 0.4) between the item and the subscale it was assumed to contain. Discriminant validity was supported if the item showed a higher correlation with its assumed subscale than the other subscales. We also conducted a correlational analysis for comparison with other outcome measurement instruments (aforementioned).

Several previous studies have reported that nulliparous women experience higher levels of PrA than parous women, especially for fear of giving birth [15,18,24]. If measurement invariance across nulliparous and parous women was supported (as described above), we performed a *t*-test to compare the mean scores of the overall scale and each subscale of the PRAQ-R2 between the two groups to evaluate known-group validity. The significance threshold was set at *p* < 0.05.

All statistical analyses were performed using R software for Windows (version 4.1.2; R Foundation for Statistical Computing, Vienna, Austria). We used the following packages: “misty” [37], “ufs” [38], “irr” [39], “polycor” [40], “lavaan” [41], and “semPlot” [42]. In the correlation analyses, Spearman’s rank correlation was used instead of Pearson’s correlation when the scores were not normally distributed. Missing values were checked for all variables. If missing data were small enough, a complete case analysis was performed for all statistical analyses.

## 3. Results

### 3.1. Content Validity

#### 3.1.1. Expert Meetings

At both meetings, members unanimously agreed on the relevance of each item to the scale construct. Regarding comprehensiveness, we found other PrA factors not in the scale through the meeting with midwives, which were “anxiety about feeding” and “social support”.

#### 3.1.2. Pilot Study through Cognitive Interviews

All interview participants completed the PRAQ-R2 (Japanese version) within 1 min and 30 s; they agreed with the comprehensibility of the instructions, items, and response options. All participants agreed that each item was relevant to their experiences. Regarding comprehensiveness, we found other PrA factors not included in the scale in these interviews: “anxiety about healthcare facilities” and “returning to work after childbirth”.

### 3.2. Validation Study

#### 3.2.1. Participant Characteristics

A total of 112 women completed the questionnaires at a median of 9 weeks of pregnancy (interquartile range (IQR): 9–11). Eight women did not return the questionnaire: three were transferred to different hospitals, two were hospitalized, and three were unknown.

Table 1 shows the demographic and obstetric characteristics of the participants who completed the questionnaire. Less than half were expecting their first baby. The majority of the participants were married. More than half of the participants had graduated from a university or higher and reported an annual income of more than JPY eight million.

#### 3.2.2. Scores Distribution of the PRAQ-R2 (Japanese Version)

**Figure 1:**
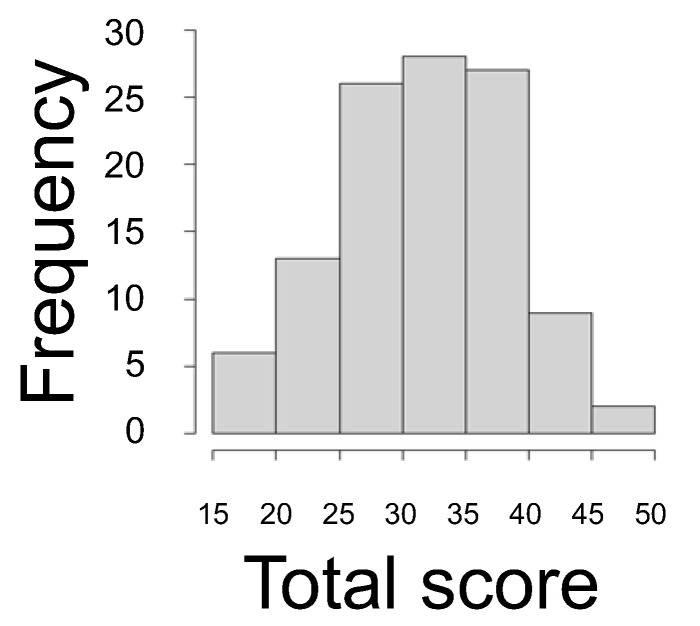
Histogram of the PRAQ-R2 (Japanese version) total score (N = 112).

Only one missing value was observed for item 5 in the PRAQ-R2 (Japanese version) in this study, supporting the adoption of complete case analysis. Figure 1 shows a histogram of the PRAQ-R2 (Japanese version) total score. The total score mean (SD) was 32.14 (6.88). The mean (SD) of each subscale was 9.80 (2.75) for FOGB, 14.2 (3.86) for WBHC, and 8.11 (2.89) for COA.

The frequency distribution and mean (SD) for each item are listed in Table A1. Most items visually assumed a skewed distribution by checking histograms, which supported the adoption of the WLSMV estimator for CFA. A ceiling effect was observed in one item (item 2); however, all response categories were used by the participants for all items, including item 2.

#### 3.2.3. Internal Consistency

Table 2 lists the internal consistency values of each item. The omega total for the overall scale of the Japanese version of the PRAQ-R2 was 0.90. The omegas for the subscales were as follows: FOGB, 0.80; WBHC, 0.94; and COA, 0.75.

#### 3.2.4. Measurement Error and Reliability

In the retest analysis, we excluded participants who answered the retest questionnaires after >14 days as an excessively long interval was considered inappropriate during an unstable early pregnancy since regular checkups were usually conducted every two weeks. A total of 27 participants answered at a mean (SD) value of 3.7 (3.7) days after the first questionnaires. Regarding measurement error, the SEM value for the total score of the Japanese PRAQ-R2 was 3.4 and the SDC was 9.5. The ICC for the total score was 0.76.

#### 3.2.5. Structural Validity

Table A2 reports the polychoric correlation matrix sorted to match the hypothesized subscales of the original scale. High correlations (ρ > 0.4) were found between the items within all hypothesized subscales.

**Figure 2:**
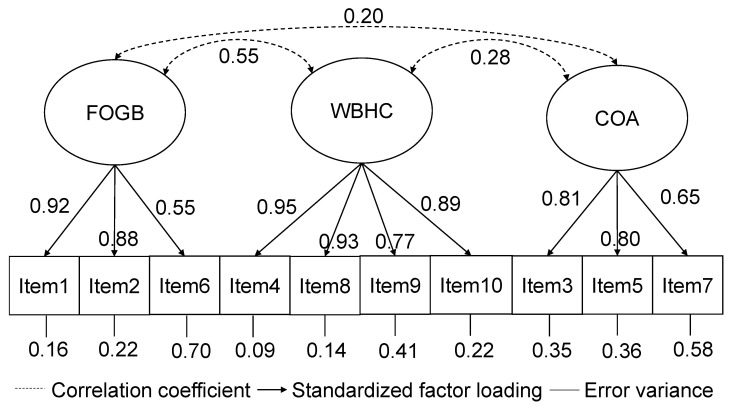
Factor structure of the Japanese version of PRAQ-R2. FOGB, fear of giving birth; WBHC, worries about bearing a physically or mentally handicapped child; and COA, concern about own appearance.

Confirmatory factor analysis of the Japanese version of the PRAQ-R2 showed an optimal-to-good fit for this sample (CFI = 0.995, TLI = 0.995, and RMSEA = 0.077). Figure 2 presents the standardized factor loadings with the correlation coefficients of the subscales. The factor loadings were high and similar to those of the original scale [15]. Item 6 had the lowest factor loading and highest error variance. Low-to-medium correlations at the subscale level confirm the three-factor model.

#### 3.2.6. Measurement Invariance across Groups (MGCFA)

The configural invariance model had a good fit (CFI = 0.997, TLI = 0.996, and RMSEA = 0.058), indicating that configural invariance was established. Second, the weak invariance model showed an optimal-to-good fit: CFI = 0.996, TLI = 0.995, and RMSEA = 0.064. The fit of the weak invariance model was not substantially worse than that of the configural model: ΔCFI = 0.001 and ΔRMSEA = −0.008, respectively, supporting weak invariance. Third, a strong invariance model showed a good fit: CFI = 0.998, TLI = 0.998, and RMSEA I = 0.036. The strong invariance model fit was better than that of the weak invariance model: ΔCFI = −0.002 and ΔRMSEA = −0.008, which supports strong invariance (both the item loadings and item intercepts were similar across the two groups). These results suggest that there were no systematic response biases and that we could compare the means of the latent variables across the two groups.

#### 3.2.7. Hypotheses Testing for Construct Validity

Table 3 reports the multitrait-scaling analysis used to compare the subscales of the Japanese version of the PRAQ-R2. All items were correlated (ρ > 0.4) with their assumed subscale: FOGB (three items), 0.47–0.70; WBHC (four items), 0.67–0.85; and COA (three items), 0.50–0.55. All items showed a lower correlation with other subscales than with their assumed subscale: FOGB, 0.07–0.49; WBHC, 0.11–0.48; and COA, 0.09–0.21.

In this study, the omega total value of the J-PSEQ subscales and HADS-A were 0.92 and 0.87, respectively. In the correlation analysis to assess convergent validity, Pearson’s correlation between the sum score of FOG and WBHC in the Japanese version of PRAQ-R2 and the sum score of two J-PSEQ subscales (“concern for well-being of self and baby” and “fear of pain, helplessness, and loss of control”) was 0.70. For discriminant validity, Spearman’s correlation between the total score of the Japanese version of the PRAQ-R2 and the HADS-A was 0.24.

As measurement invariance across nulliparous and parous women was supported by the MGCFA, we compared the mean scores of the total and each subscale of the PRAQ-R2 (Japanese version) between the two groups using a *t*-test (Table 4). The mean total score was significantly higher in the nulliparous than the parous women. Among the subscales, particularly the mean FOGB score was higher in nulliparous than parous women.

## 4. Discussion

We developed a Japanese version of the PRAQ-R2, which is a measure for the assessment of pregnancy-specific anxiety. Following cognitive interviews, we conducted a validation study of this new instrument among Japanese women in their first trimester of pregnancy and demonstrated its validity and reliability.

Item 2 (worry about pain) exhibited a ceiling effect. In Japan, labor pain is traditionally considered a natural part of birth processes and is necessary for the transition to motherhood; a previous study found that some primiparous women believed that experiences like enduring pain lead to greater confidence as a mother [43]. Their perceived mission to endure pain without pain relief to become a good mother may result in a ceiling effect of worrying about pain in the Japanese culture. Although one item yielded a ceiling effect, this new scale could detect small differences between the nulliparous and parous groups at a statistically significant level in a small sample, which indicates that this scale has sufficient sensitivity. We do not believe that there is a significant impact of a ceiling effect for a single item. In addition, all response categories were used by the participants in item 2, scaling success for convergent and discriminant validity in multitrait-scaling analysis, and the decrease in omega total value when removing item 2 indicated sufficient functionality of this item. Therefore, we decided not to exclude item 2.

The item–total correlation showed that none of the items had particularly weak or strong correlations. The omega value for the overall scale was 0.90, indicating excellent internal consistency. The omega values when each item was excluded were 0.71–0.82; therefore, the omega of the complete scale was never exceeded. Because no item lacked internal consistency, we decided not to exclude any items from the perspective of internal consistency.

The ICC score in this study showed good reliability, indicating that the Japanese version of the PRAQ-R2 could discriminate between women in the sample. Based on the SDC value (9.5), changes in the total scores by more than ten points before and after the future intervention may be interpreted as a change beyond measurement error.

The same three-factor structure as in the original version was supported by the Japanese sample. Confirmatory factor analysis showed a satisfactory model fit, which is in line with the results from a validation study of the original PRAQ-R2 [15]. Factor loadings of the individual items on their contained factors were very similar to those of the original version [15], indicating the high factorial validity of the developed Japanese version of the PRAQ-R2. As presented in the original version [15] and in a German validation study [25], item 6 (worry about losing control) showed the lowest factor loading and highest error variance. Although the model fit without this item was slightly better, a previous study included this item in the scale by reporting the association between symptoms of social phobia and this item [25]. We will also keep the same 10-item scale structure to facilitate future comparisons with other countries, and item 6 may be relevant in women experiencing specific forms of anxiety.

To test the construct validity hypotheses, we conducted three analyses. First, the multitrait-scaling analysis showed that all items had a higher correlation coefficient in convergent than discriminant correlation. Second, the sum score of the FOG and WBHC in the Japanese version of the PRAQ-R2 was strongly correlated with the sum score of the two subscales of the J-PSEQ, supporting convergent validity. In addition, only a weak correlation between the total score of the Japanese version of the PRAQ-R2 and HADS-A supported the discriminant validity of this scale for generalized anxiety disorders. The current study indicates that this new scale can capture pregnancy-specific anxiety and may have the potential to predict perinatal adverse outcomes. Third, to evaluate known-group validity, we compared scores on this scale between nulliparous and parous women. This study confirmed measurement invariance between nulliparous and parous women through MGCFA and indicated that nulliparous women have a greater level of anxiety than parous women regarding fear of giving birth, which is consistent with previous studies [15,18,24]. This may be a normative reaction to the women’s first labor experience occurring in the near future. This result indicates that further support for fear of childbirth is needed, especially for primiparous women. Through three analyses, all hypotheses were supported, and the construct validity of this new scale was demonstrated.

This study has several limitations. First, there may have been selection bias in cognitive interviews and validation study. Although similar to those reported in the Japanese national data on age, marital status, and means of conception, the nonprobability samples in this study were rather homogenous in terms of socioeconomic background, possibly because this study was conducted in an urban area. Although financial incentive was reasonable and the majority of participants reported their annual income higher than national averages in Japan, we cannot rule out the possibility that the reimbursement also induced selection bias. Second, owing to its cross-sectional design, we could not evaluate the responsiveness or predictive validity of the scale. Further longitudinal studies will be needed to examine the intrapersonal changes and predictive validity of this scale. Third, although we determined and achieved the sample size in accordance with COSMIN guidelines, it did not reach an adequate level in the retest analysis. To our knowledge, the measurement error of this scale was first reported in this study; however, it may be difficult for women in the early stages of pregnancy to answer the retest within adequate intervals, resulting in a sample size below expectation. Fourth, the impact of other PrA elements extracted from the cognitive interviews remains unknown. There is scope to investigate whether these elements can predict outcomes among Japanese women.

Despite these limitations, the translated Japanese version of the PRAQ-R2 developed in this study is the first reliable and validated instrument to assess PrA in Japan, with evidence of its psychometric properties.

## 5. Conclusions

The Japanese version of the PRAQ-R2 is a reliable measure for the assessment of PrA in a Japanese population and is able to capture the intricacies of pregnancy-specific anxiety. This study confirmed the validity of the three-factor PRAQ-R2 in a sample of Japanese women in their first trimester of pregnancy. It can be used for the assessment of PrA regarding child health, child loss, childbirth, and body image, and for facilitating screening and intervention in both nulliparous and parous women. This study will help researchers examine the associations and processes between PrA and psychological and physical health and will aid healthcare professionals in identifying PrA in a medical setting.

## Figures and Tables

**Table 1:** Demographic and obstetric characteristics of participants (N = 112).

Variables	N	%
Age (years), mean (SD ^1^)	33.0 (3.9)	
Body mass index before pregnancy (kg/m^2^), median (IQR ^2^)	21.0 (19.0–22.3)	
Parity		
Nulliparous	49	44
Previous abortion		
Yes	28	25
Marital status		
Non-single	110	98
Having a paid job	93	83
Full-time	87	78
Education		
Junior high/high school	8	7
Technical/junior college	31	28
University or higher	73	65
Income		
JPY <4.0 million ^3^	11	10
JPY 4.0–5.9 million	19	17
JPY 6.0–7.9 million	20	18
JPY 8.0–10 million	26	23
JPY >10 million	36	32
Means of conception		
Natural	101	90
Artificial insemination	3	3
In vitro fertilization	8	7

^1^ standard deviation; ^2^ interquartile range; ^3^ USD 1 is roughly equivalent to JPY 100 JPY.

**Table 2:** Contents and internal consistency values of each item (N = 112).

Item	Contents	Item–Total Correlation ^1^	Omega Total, If Item Removed
Fear of giving birth			
1	anxious about delivery	0.65	0.71
2	worry about pain	0.63	0.78
6	worry about losing control	0.50	0.80
Worries about bearing a handicapped child			
4	child in poor health	0.77	0.73
8	child mentally handicapped	0.73	0.75
9	perinatal death of child	0.63	0.76
10	physical defect of child	0.69	0.74
Concern about appearance			
3	not regaining figure	0.58	0.80
5	unattractive appearance	0.46	0.80
7	weight gain	0.42	0.82

^1^ Spearman’s correlation coefficient.

**Table 3:** Multitrait-scaling analysis between items and subscales on the Japanese version of the PRAQ-R2 (N = 112).

Item	FOGB ^1^	WBHC ^2^	COA ^3^
1	0.67 ^4^	0.49	0.07
2	0.70 ^4^	0.40	0.15
6	0.47 ^4^	0.17	0.25
4	0.48	0.80 ^4^	0.22
8	0.41	0.76 ^4^	0.21
9	0.32	0.67 ^4^	0.13
10	0.35	0.85 ^4^	0.11
3	0.18	0.21	0.54 ^4^
5	0.13	0.16	0.55 ^4^
7	0.09	0.09	0.50 ^4^

^1^ fear of giving birth; ^2^ worries about bearing a physically or mentally handicapped child; ^3^ concern about own appearance; ^4^ corrected for overlap.

**Table 4:** Comparison of the scores on the Japanese version of the PRAQ-R2 between nulliparous and parous women (N = 112).

PRAQ-R2	Mean (SD ^1^)		*t*	*p*-Value
	Nulliparous (N = 49)	Parous (N = 63)		
Total	34.5 (6.1)	30.3 (7.0)	3.34	0.001
FOGB ^2^	11.3 (2.1)	8.7 (2.7)	5.80	<0.001
WBHC ^3^	15.1 (3.7)	13.5 (3.9)	2.24	0.027
COA ^4^	8.0 (2.8)	8.2 (2.9)	−0.41	0.68

^1^ standard deviation; ^2^ fear of giving birth; ^3^ worries about bearing a physically or mentally handicapped child; ^4^ concern about own appearance.

## Data Availability

The data presented in this study are available on request from the corresponding author. The data are not publicly available for ethical reasons.

## Supplementary Materials

The following supporting information can be downloaded at: https://www.mdpi.com/article/10.3390/healthcare11131935/s1, Table S1: Participant characteristics of cognitive interviews (N = 6); Table S2: Semistructured questions in the cognitive interview.

## Appendix A

**Table A1:** The frequency distribution of each item of the Japanese version of PRAQ-R2 (N = 112).

	Item 1	Item 2	Item 3	Item 4	Item 5
Histogram					
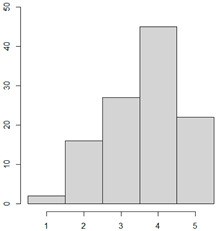
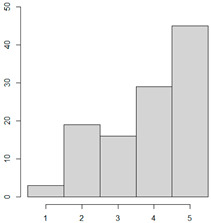
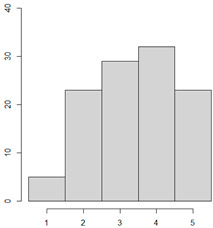
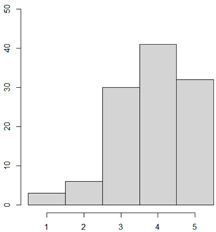
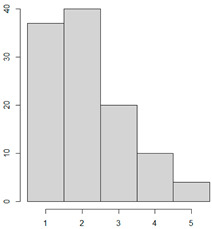
Mean	3.61	3.84	3.40	3.83	2.14
SD ^1^	1.02	1.20	1.16	0.99	1.09
	Item 6	Item 7	Item 8	Item 9	Item 10
Histogram					
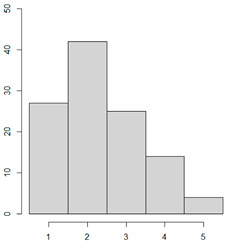
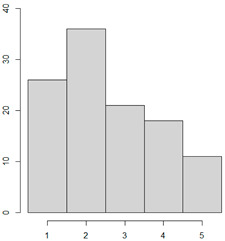
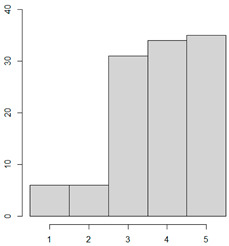
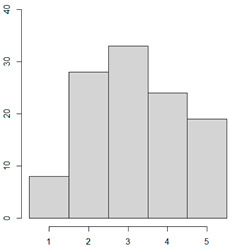
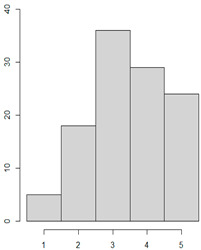
Mean	2.34	2.57	3.77	3.16	3.44
SD	1.09	1.28	1.11	1.19	1.13

^1^ standard deviation.

## Appendix B

**Table A2:** Polychoric correlation matrix of the Japanese version of the PRAQ-R2 items (N = 112).

**Item**	**1**	**2**	**6**	**4**	**8**	**9**	**10**	**3**	**5**	**7**
1	1									
2	0.81	1								
6	0.46	0.56	1							
4	0.55	0.53	0.23	1						
8	0.44	0.46	0.20	0.91	1					
9	0.43	0.26	0.27	0.62	0.58	1				
10	0.48	0.34	0.20	0.80	0.79	0.78	1			
3	0.14	0.12	0.25	0.28	0.21	0.28	0.24	1		
5	−0.002	0.13	0.25	0.23	0.29	0.10	0.12	0.61	1	
7	−0.04	0.14	0.16	0.16	0.19	0.004	−0.04	0.50	0.57	1
