# Supplementary material for: Development of the Japanese Version of Pregnancy-Related Anxiety Questionnaire—Revised-2: Measurement and Psychometric Properties
Source: Healthcare (Basel). 2023 Jul 4;11(13):1935. doi: 10.3390/healthcare11131935 (PMC10341067; doi:10.3390/healthcare11131935)
Supplement: Supplementary file 1 [file healthcare-11-01935-s001.zip › Table S1.pdf]

Table S1. Participant characteristics of cognitive interviews (N=6).

Age (years)	GA (weeks)	Facility	Number of deliveries	Number of abortions	IVF	Job	Education	Economic comfort	Participating prenatal class	Risk
34	31	Hospital	0	0	Yes	Full-time worker	University	Some	Yes	NA
29	24	University hospital	0	0	No	Full-time worker	University	Not much	No	Been abused
33	33	Clinic	0	2	Yes	Contract worker	University	Not much	Yes	Fibroid
36	23	Hospital	1	3	No	Full-time worker	University	Not much	No	NA
27	31	Clinic	0	0	No	Full-time worker	University	Not much	Yes	NA
36	26	Hospital	0	0	No	Full-time worker	Technical school	Some	Yes	NA

GA, gestational age; IVF, in vitro fertilization.
