# Supplementary material for: Development of the Japanese Version of Pregnancy-Related Anxiety Questionnaire—Revised-2: Measurement and Psychometric Properties
Source: Healthcare (Basel). 2023 Jul 4;11(13):1935. doi: 10.3390/healthcare11131935 (PMC10341067; doi:10.3390/healthcare11131935)
Supplement: Supplementary file 1 [file healthcare-11-01935-s001.zip › Table S2.pdf]

Table S2. Semi-structured questions in the cognitive interview.

Topics	Questions
Instructions	Were the instructions easy to understand?
Response options	Were the response options easy to understand?
Each item	How did you feel when asked this question?
	Was this question difficult to answer?
	Did you feel distressed, confused, or flustered?
	Is this question relevant to your pregnancy or treatment?
Overall scale	How did you feel about answering this questionnaire?
	Can you think of any other important items to include?
	Do you have any other comments about this questionnaire?
